# Deciphering Probabilistic Species Interaction Networks

**DOI:** 10.1111/ele.70161

**Published:** 2025-06-26

**Authors:** Francis Banville, Tanya Strydom, Penelope S. A. Blyth, Chris Brimacombe, Michael D. Catchen, Gabriel Dansereau, Gracielle Higino, Thomas Malpas, Hana Mayall, Kari Norman, Dominique Gravel, Timothée Poisot

**Affiliations:** ^1^ Département de Sciences Biologiques Université de Montréal Montreal Quebec Canada; ^2^ Département de Biologie Université de Sherbrooke Sherbrooke Quebec Canada; ^3^ Quebec Centre for Biodiversity Science Quebec Canada; ^4^ School of Biosciences The University of Sheffield Sheffield UK; ^5^ Department of Ecology and Evolutionary Biology University of Toronto Toronto Ontario Canada

**Keywords:** ecological modelling, ecological networks, food webs, host–parasite interactions, metaweb, sampling, spatial scale, species interactions, temporal scale, uncertainty

## Abstract

Representing species interactions probabilistically as opposed to deterministically conveys uncertainties in our knowledge of interactions. The sources of uncertainty captured by interaction probabilities depend on the method used to evaluate them: uncertainty of predictive models, subjective assessment of experts, or empirical measurement of interaction spatiotemporal variability. However, guidelines for the estimation and documentation of probabilistic interaction data are lacking. This is concerning because our understanding of interaction probabilities depend on their sometimes elusive definition and uncertainty sources. We review how probabilistic interactions are defined at different spatial scales. These definitions are based on the distinction between the realisation of an interaction at a specific time and space (local networks) and its biological or ecological feasibility (metaweb). Using host–parasite interactions in Europe, we illustrate how these two network representations differ in their statistical properties, specifically: how local networks and metawebs differ in their spatial and temporal scaling of interactions. We present two approaches to inferring binary interactions from probabilistic ones that account for these differences and show that systematic biases arise when directly inferring local networks from metawebs. Our results underscore the importance of more rigorous descriptions of probabilistic species interactions that specify their conditional variables and uncertainty sources.

## Graphical abstract

We review how probabilistic species interactions are defined at different spatial scales. These definitions are based on the distinction between the realisation of an interaction at a specific time and space (local networks) and its biological or ecological feasibility (metaweb). Using host–parasite interactions in Europe, we illustrate how these two network representations differ in their statistical properties, specifically: how local networks and metawebs differ in their spatial and temporal scaling of interactions.

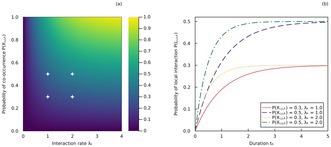

## 1 Introduction

### 1.1 Species Interactions Are Variable and Uncertain

As we navigate global biodiversity change, filling in knowledge gaps about biodiversity becomes instrumental to monitoring and mitigating those changes (Hortal et al. 2015; Gonzalez and Londoño 2022; Abrego et al. 2021). However, cataloguing species, populations and, in particular, ecological interactions (e.g., predation, parasitism and pollination) is challenging (Polis 1991; Pascual and Dunne 2006). There are methodological and biological constraints that hinder our ability to document species interactions, leading to uncertainty in our knowledge of interactions. For example, the spatial and temporal uncoupling of species (e.g., nocturnal and diurnal species coexisting in the same space, Jordano 1987) and the large number of rare and cryptic interactions in a community contribute to these knowledge gaps by making it more difficult to observe interactions (Jordano 2016).

Several conditions must be satisfied for an interaction to be realised locally. First, both species must have overlapping geographic ranges, that is, they must co‐occur (Morales‐Castilla et al. 2015; Cazelles et al. 2016). Second, they must have a non‐zero probability of meeting (Poisot et al. 2015). Probabilities of interspecific encounters are typically low, especially for rare species (Vázquez et al. 2007; Canard et al. 2012, 2014). The probability that species meet also depends on their biology, such as their phenology (Olesen et al. 2010; Singer and McBride 2012) and discoverability (Broom and Ruxton 2005). Finally, when species encounter, an interaction occurs only if their traits, such as their phenotypes (Bolnick et al. 2011; Stouffer et al. 2011; Gravel et al. 2013) and behaviour (Pulliam 1974; Choh et al. 2012), are locally compatible (Poisot et al. 2015). Because these conditions are not consistently met locally, there will inevitably be instances where interactions will occur and others where they will not.

Documenting the location and timing of interactions is difficult when accounting for their spatiotemporal variability (Poisot et al. 2012, 2015). Knowing the biological capacity of two species to interact is necessary but not sufficient for inferring their interaction at a specific time and space. Environmental factors, such as temperature (Angilletta Jr. et al. 2004), drought (Woodward et al. 2012), climate change (Gilman et al. 2010; Woodward et al. 2010; Araujo et al. 2011), habitat characteristics (e.g., presence of refuges where prey can hide from predators, Grabowski 2004) and land use change (Tylianakis et al. 2007), contribute to this spatiotemporal variability by impacting species abundance and traits. Interactions may also be influenced by a third species (e.g., a more profitable prey species, Golubski and Abrams 2011; Sanders and van Veen 2012). Even under favourable circumstances, there remains a possibility that the interaction does not occur locally, either due to the intricate nature of the system or simply by chance. If it does occur, it might go undetected, particularly if it happens infrequently. In this context, it is unsurprising that our knowledge of ecological interactions remains limited (Hortal et al. 2015) despite extensive biodiversity data collection (Schmeller et al. 2015).

**FIGURE 1:**
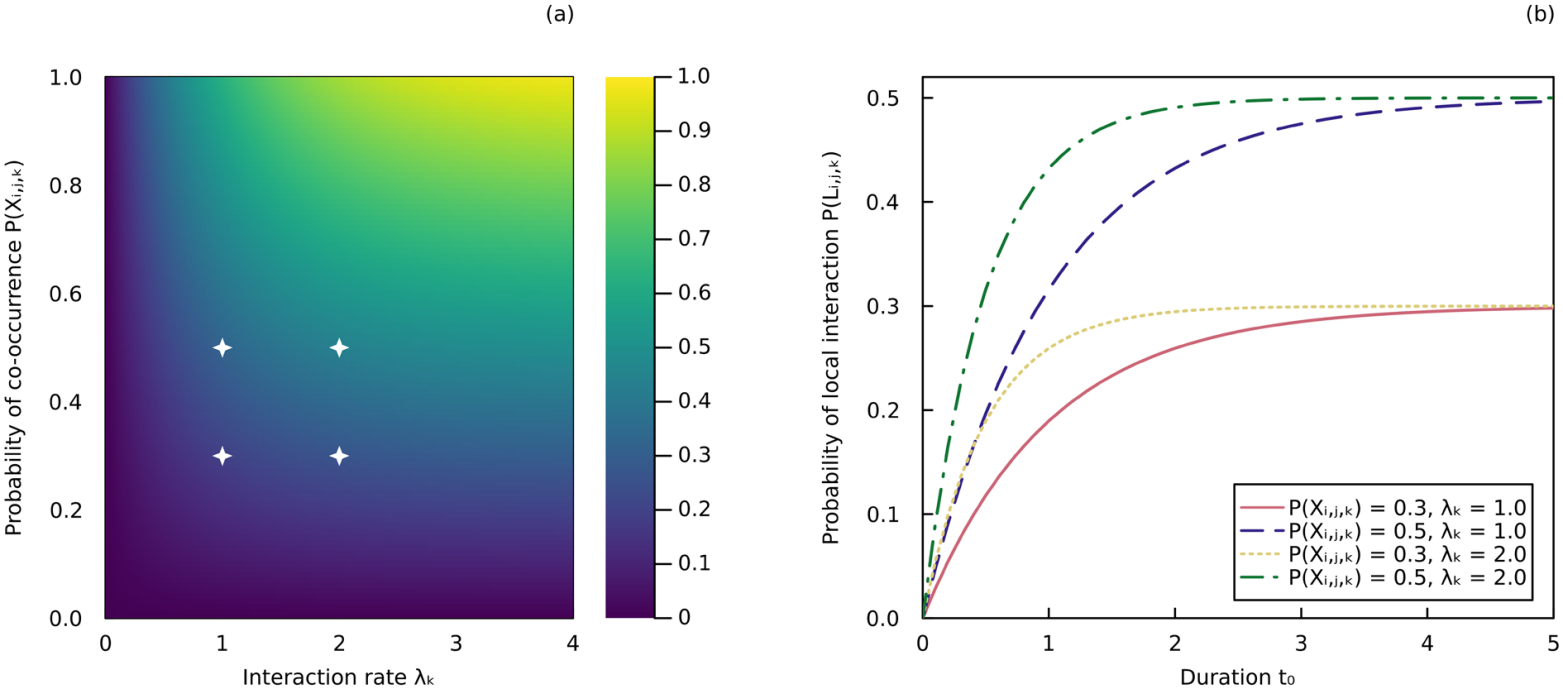
Parameters of the spatiotemporally explicit model of interactions. (a) Probability of local interaction $\varphi_{i,j,k}=P\left(L_{i,j,k}\right)$ (short for $P\left(L_{i,j,k}=1\right)$) given by the process model (Equation (15), Box 1) under different values of $\lambda_{i,j,k}$ (interaction rate) and $P\left(X_{i,j,k}\right)$ (probability of co‐occurrence, short for $P\left(X_{i,j,k}=1\right)$), with $t_{0}=1$ (duration). The probability of local interaction represents the probability that the two taxa will interact at least once within the given time interval. Parameters $t_{0}$ and $\lambda_{i,j,k}$ have complementary units (e.g., $t_{0}$ in months and $\lambda_{i,j,k}$ in number of interactions per month). The parameter values used in the right panel are denoted by the white stars. (b) Scaling of the probability of interaction with the duration parameter $t_{0}$, for different values of $\lambda_{i,j,k}$ and $P\left(X_{i,j,k}\right)$.

We distinguish two types of uncertainty: the uncertainty arising from interaction variability (aleatory uncertainty) and the uncertainty due to incomplete knowledge (epistemic uncertainty, Walker et al. 2003). Interaction variability is defined as the changes in the occurrence or strength of interactions along spatial, temporal, or environmental axes (Poisot et al. 2015). For instance, the proportion of networks in which an interaction occurs is a measure of interaction variability. In contrast, knowledge uncertainty represents our lack of knowledge about parameters and variables (e.g., not knowing whether an interaction occurs or not). When using statistical models to infer interactions, sources of knowledge uncertainty include input data, parameter and model structure uncertainties (Simmonds et al. 2024). Input data uncertainty arises from our inability to observe all interactions and from measurement errors in environmental and biological variables used for inference. Parameter uncertainty represents a plausible range of values for a parameter whose exact value is unknown. For example, we may calculate a range of values for interaction variability (e.g., there could be a 50% certainty that an interaction occurs 50% of the time). When interaction variability is used as a model parameter (Box 1, Figure 1), its degree of accuracy may be determined by parameter uncertainty. Model structure uncertainty recognises that different models may adequately predict interactions. Simmonds et al. (2024) underscores the importance of quantifying and reporting these sources of uncertainty, and propagating them to model output (such as predicted interactions) and higher‐level measures (such as network structure). The distinction between variability and knowledge uncertainty (hereafter referred to as uncertainty) is important because uncertainty can be reduced by collecting more data, but not variability. Moreover, uncertainty is typically represented by probability distributions, whereas variability is often modelled using frequency distributions. This distinction allows us to better understand the sources of our knowledge gaps about ecological interactions.

BOX 1 **A spatiotemporally explicit model of interactions.**

Ecologists may use predictive models to reconstruct local networks across time and space. We introduce and develop a generative Bayesian model for local interactions which explicitly accounts for their spatiotemporal variability. Our model is not designed for regional interactions, which do not vary spatially nor temporally. Rather, it could prove valuable for generating new data on local interactions across time and space, following parameter inference.

As indicated by Equation (2), the probability that two taxa i and j interact locally can be obtained by multiplying their probability of interaction given co‐occurrence with their probability of co‐occurrence. The probability of interaction given co‐occurrence can be made temporally explicit by modelling it as a Poisson process, that is, an event that occurs at a constant average rate $\lambda_{i,j,k}$. This parameter represents the local expected frequency of interaction between co‐occurring taxa (i.e., a measure of the temporal variability of the interaction). The exponential distribution gives us the probability density for the amount of time that passes between two consecutive realisations of an event with a constant average rate. Therefore, the cumulative density function of the exponential distribution can be used to model the probability that two co‐occurring taxa will interact at least once during a time interval $t_{0}$:

(13) $$P\left(L_{i,\;j,\;k}=1|X_{i,\;j,\;k}=1\right)=1-e^{-\lambda_{i,\;j,\;k}t_{0}},$$

which tends toward 1 as $t_{0}\to \infty$ if $\lambda_{i,\;j,\;k}>0$. In other words, two co‐occurring taxa with a nonzero rate of interaction will inevitably interact at least once in a sufficiently long time interval.

The occurrence of an interaction between i and j may be the result of a Bernoulli trial with parameter $\varphi_{i,j,k}$ representing the probability of interaction $P\left(L_{i,\;j,\;k}=1\right)$. A Bayesian model can be built using the preceding equations to generate new interaction data, following the inference of $\lambda_{i,\;j,\;k}$ and $P\left(X_{i,\;j,\;k}\right)$.

(14) $$L_{i,\;j,\;k}∼\text{Bernoulli}\left(\varphi_{i,\;j,\;k}\right)$$

(15)

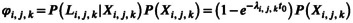

(16) $$P\left(X_{i,\;j,\;k}\right)∼\text{Beta}\left(2,\;2\right)$$

(17) $$\lambda_{i,\;j,\;k}∼\text{Exponential}\left(2\right)$$

In Figure 1, we show the variation in the probability of interaction under different parameter values. In the right panel, we notice that the probability of interaction always converges toward the probability of co‐occurrence $P\left(X_{i,j,k}=1\right)$, for all positive values of the interaction rate. This suggests that, over time, the probability that two taxa interact is given by their probability of co‐occurrence, assuming they have the biological capacity to interact under suitable environmental conditions.

Spatiotemporally explicit models of interactions have a wide range of applications, including predictions, research design, and sampling evaluation. They can be customised in different ways, such as by linking parameters to specific environmental or biological variables. For instance, the probability of co‐occurrence could be modelled as a function of climatic variables, while the interaction rate could be modelled based on taxa abundances. This could enable better predictions of interactions across space and time based on occurring environmental and biological conditions. Additionally, these models can help determine the sampling duration needed to obtain a specific probability of observing an interaction, thereby informing the evaluation of sampling completeness.

### 1.2 Species Interactions as Probabilistic Objects

The recognition of the variability and uncertainty of species interactions has led ecologists to expand their representation of ecological networks to include a probabilistic view of interactions (Poisot et al. 2016; Dallas et al. 2017; Fu et al. 2021). This allows filling in the Eltonian shortfall (i.e., the gap between current knowledge and a comprehensive understanding of interactions, Hortal et al. 2015) by modelling the probability of occurrence of interactions. This can be important for directing efforts and taking action (Carlson et al. 2021), especially in places where access and resources for research are scarce. A probability is a measure of how likely a specific outcome is, based on both the uncertainty and variability of interactions. Interaction probabilities may be uncertain when there is a distribution of plausible probability values. Probabilistic interactions have been applied to direct interactions, which are conceptually and mathematically analogous regardless of their biological type (e.g., predation and pollination). This is in contrast with indirect interactions (e.g., competition), which arise from distinct ecological processes and are often not directly observable (Kéfi et al. 2015, 2016). By accounting for the uncertainty and variability of direct interactions, networks of probabilistic interactions (which differ from *probabilistic networks* measuring the probability of the *whole* network) may provide a more realistic portrait of species interactions.

Networks of probabilistic interactions, within a Bayesian perspective, express our degree of belief (or confidence) regarding the feasibility, occurrence or observation of interactions. In a frequentist approach, they represent the expected relative frequencies of interactions over many repeated trials or sampling events. Our level of confidence should be more definitive (approaching either 0 or 1) as we extend our sampling to a broader area and longer duration, thereby diminishing knowledge uncertainty (but not the estimation of interaction variability). In contrast, interactions are simply regarded as either occurring or not in networks of deterministic binary interactions. In the broadest sense, binary interactions are a type of probabilistic interaction, in which the numerical value is restrained to 0 (non‐occurring) or 1 (occurring). In networks of probabilistic interactions, only forbidden interactions (i.e., interactions prohibited by biological traits or species absence, Jordano et al. 2003; Olesen et al. 2010) have a probability of zero. Understanding the nuances between probabilistic and binary interactions is essential for accurately modelling and interpreting ecological networks.

The development and application of computational methods in network ecology, often based on a probabilistic representation of interactions, can alleviate (and guide) the sampling efforts required to document species interactions (Strydom et al. 2021). For example, statistical models can be used to estimate the probability of missing (false negatives) and spurious (false positives) interactions (Guimerà and Sales‐Pardo 2009), helping us identify places where sampling is most needed to reduce uncertainty. Statistical models can predict networks without prior knowledge of interactions, for example, using body size (Petchey et al. 2008; Gravel et al. 2013; Caron et al. 2024), phylogeny (Elmasri et al. 2020; Strydom et al. 2022), or a combination of niche and neutral processes (Bartomeus et al. 2016; Pomeranz et al. 2019). Before being used to test ecological hypotheses, predicted networks must be validated against empirical data (Brimacombe et al. 2024), which could be sampled strategically to optimise the validation process. Topological null models, which generate networks of probabilistic interactions by preserving chosen characteristics of the adjacency matrix while intentionally omitting others (Bascompte et al. 2003; Fortuna and Bascompte 2006), are examples of common probabilistic models. Null models can produce underlying distributions of network measures for null hypothesis significance testing. However, how the uncertainty of interactions propagates to network structure remains to be elucidated. Many measures describe the structure (Poisot et al. 2016) and diversity (Ohlmann et al. 2019; Godsoe et al. 2022) of probabilistic interaction networks. These models and measures support the use of probabilistic interactions for studying a wide range of ecological questions, from predicting species distributions (Cazelles et al. 2016) to forecasting the impact of climate change on ecological networks (Gilman et al. 2010).

### 1.3 We Lack a Clear Understanding of Probabilistic Species Interactions

We still lack a precise definition of probabilistic interactions, which makes the estimation and use of these data more difficult. We take a step back by outlining different ways in which probabilistic interactions are defined and used in network ecology. We distinguish two broad categories of probabilistic interaction networks: local networks describing probabilities of realised interactions, and regional networks (metawebs) describing probabilities of potential interactions. Potential interactions are defined as the biological or ecological capacity of taxa to interact (i.e., the probability that they interact if they were to encounter each other, given sufficient time and appropriate environmental conditions) whereas realised interactions are their occurrence in a well‐defined space and time. For two co‐occurring taxa and over enough time, the probability of local interaction tends toward the probability of regional interaction, a longer duration increasing the probability that these taxa will eventually encounter each other and that suitable environmental conditions will occur. We compare these two network representations and examine their properties and relationships with space and time.

The lack of guidelines on probabilistic interaction data is worrisome, as it affects both data producers and re‐users who generate and manipulate these numbers. This is concerning because decisions regarding network construction can affect our understanding of network properties (Brimacombe et al. 2023). There is currently no reporting standard that could guide the documentation of all types of probabilistic interactions (Salim et al. 2022 discuss data standards for deterministic mutualistic networks). We discuss general best practices (such as thorough data documentation) rather than recommending specific data formats or software tools, because the choice of data formats depends on the variable, research question and software used, while different tools (e.g., Stan, Julia) can be used for making inference. Data documentation should outline the scale (local or regional) and biological type (e.g., predatory or pollination) of interactions, the taxonomic level and characteristics (e.g., life stages) of the individuals involved, the mathematical expression of probabilities, including clearly identified conditional variables (e.g., area, duration and environmental conditions) and the model used. Thorough data documentation helps adequately interpret and manipulate probabilistic interaction data. In the following sections, we show why this is important by comparing different types and conditions of probabilistic interactions as we scale up from pairwise interactions to interactions within local and regional networks.

## 2 Pairwise Interactions: The Building Blocks of Ecological Networks

### 2.1 What Are Probabilistic Interactions?

Consider a scenario where an avian predator has just established itself in a northern habitat home to a small rodent. Suppose their interaction has not been previously observed, either because they never co‐occurred before or because previous sampling failed to detect an interaction. What is the probability that the rodent is part of the predator's diet? This question can be answered in different ways. We could calculate the probability that their traits match, that is, that the predator has the biological attributes to capture and consume the rodent (regional interaction). We could also calculate the probability that their traits support an interaction under the typical environmental conditions of the new habitat (also a regional interaction). For example, because avian predators hunt by sight, predation could be highly improbable when snow is present. Finally, we could calculate the probability that the avian predator will consume the rodent at *that* particular location (local interaction). Our estimation hinges on our understanding of these probabilities and the ecological processes we aim to capture.

We use the terms *metaweb* (Dunne 2006) to designate regional networks of potential interactions and *local networks* (Poisot et al. 2012) for those of realised interactions. Metawebs are the network analogues of the species pool, where local networks originate from a subset of both species and interactions of the metaweb (Saravia et al. 2022). Local interactions are the subset of all potential interactions that were either observed or predicted to occur at a given time and location. Without clear documentation, it can be challenging to know if probabilistic interactions are local or regional. A better understanding of probabilistic interactions would facilitate a more adequate use of these data and prevent misinterpretations of their biological meaning.

### 2.2 What Is the Outcome of Probabilistic Interactions?

#### 2.2.1 The Outcome of Probabilistic Interactions Is Usually Binary

Local networks and metawebs are made of nodes and edges that may be represented at different levels of organisation. The basic units of ecological networks are individuals that interact with each other (e.g., by predation, Elton 2001), forming individual‐based networks (Melián et al. 2011). The aggregation of these individuals into more or less homogeneous groups (e.g., populations, species, feeding guilds) allows us to represent nodes at broader taxonomic scales, which affects our interpretation of network properties (Guimarães 2020; Hemprich‐Bennett et al. 2021).

Ecologists have traditionally represented interactions as binary objects that were considered realised after observing at least one individual from group i interact with at least another from group j. In an adjacency matrix B of binary interactions, the presence or absence of an interaction $B_{i,j}$ can be viewed as the result of a Bernoulli trial $B_{i,j}∼\text{Bernoulli}\left(\varphi_{i,j}\right)$, with $\varphi_{i,j}=P\left(B_{i,j}=1\right)$ being the probability of interaction characterising our limited knowledge and/or interaction variability. Interaction probability may be estimated using predictive models or expert (prior) knowledge about the interaction. In networks of probabilistic interactions, the edge values $P\left(B_{i,j}=1\right)$ (which we denote as $P\left(B_{i,j}\right)$ for simplicity and better readability) are probabilistic events whose only two possible outcomes are the presence ($B_{i,j}=1$) or absence ($B_{i,j}=0$) of an interaction. Depending on the type of network (local or regional), stochastic parameters like $P\left(B_{i,j}\right)$ can be linked to environmental and biological factors such as species abundances, traits, area and time, for example, using logistic regression. This allows us to model the probability that at least two individuals interact under these conditions.

The variability of an interaction determines the number of networks in which it occurs. This number can be predicted using a Binomial distribution, assuming a constant interaction probability and independence between networks. When accounting for uncertainties in the estimation of $P\left(B_{i,j}\right)$, a Beta distribution $\text{Beta}\left(\alpha ,\beta \right)$ may be used to represent the relative likelihood of different probability values. The α and β parameters estimate the number of successes (sampled networks with the interaction) and failures (sampled networks without the interaction), respectively. The Beta distribution predicts the probability of interaction in a local network containing both species. If we consider this uncertainty, a Beta‐Binomial distribution can be used to predict the number of networks in which the interaction occurs. Empirically observing this interaction provides important information updating previous estimates of $P\left(B_{i,j}\right)$. By sampling interactions in different contexts, we can estimate their local variability more precisely.

#### 2.2.2 The Outcome of Probabilistic Interactions May Be Quantitative

Even though binary interactions constitute a valuable source of information (Pascual and Dunne 2006), they overlook interaction strengths. Represented in a quantitative adjacency matrix W, interaction strengths describe energy flows, demographic impacts or frequencies of interactions (Berlow et al. 2004; Borrett and Scharler 2019), with $W_{i,j}$ being a natural N or real R number depending on the measure. For example, they may represent local interaction rates (e.g., the flower‐visiting rates of pollinators, Herrera 1989). Relative frequencies of interactions may be used as a measure of both the strength and probability of local interactions. When interaction strengths characterise predation pressure on prey, they can serve as parameters in a Lotka–Volterra model (e.g., Emmerson and Raffaelli 2004). The extra amount of information in quantitative networks typically comes at a cost of greater sampling effort and data volume (Strydom et al. 2021), especially when quantifying the uncertainty and variability of quantitative interactions (Berlow et al. 2004). However, if two taxa are repeatedly found together without interacting, there may be more uncertainty about the occurrence of the interaction than its strength (which would assuredly be close to 0).

Like binary interactions, the uncertainty and variability of interaction strengths can be represented probabilistically. Interaction strengths can follow different probability distributions depending on the measure. For instance, they can follow a Poisson distribution $W_{i,j}∼\text{Poisson}\left(\lambda_{i,j}t_{0}\right)$ when predicting the number of times individuals interact during a time interval $t_{0}$, with $\lambda_{i,j}$ being the expected rate of interaction. The Poisson distribution can be 0‐inflated when modelling non‐interacting taxa, which constitute the majority of taxa pairs in most networks (Jordano 2016). Regardless of the measure, estimating the uncertainty of quantitative interactions enables us to consider a range of possible values of interaction strength.

Because binary interactions are usually easier to sample (Jordano 2016) and predict (Strydom et al. 2021) than quantitative interactions, they have been more frequently studied and used. Software like Ecopath (Christensen and Pauly 1992) simplifies the prediction of quantitative interactions, but the number of biological parameters that they require may hinder their application in many systems. Moreover, most published probabilistic interaction networks (e.g., Strydom et al. 2022) and methods (e.g., Poisot et al. 2016) involve probabilistic interactions with binary outcomes. This underlines the need for better guidelines on the interpretation and manipulation of probabilistic interactions with binary outcomes first. For these reasons, our primary focus is on the interpretation of interaction probabilities that determine the presence or absence of interactions, in both local networks and metawebs.

## 3 Local Networks: Communities Interacting in Space and Time

### 3.1 What Are Local Probabilistic Interactions?

Local networks of probabilistic interactions describe how likely taxa are to interact in a given local context. In local networks, edges commonly represent the probability that interactions are realised in nature, but can also represent the probability of empirically observing this interaction. Realised interactions occur locally without necessarily being observed (two locally interacting taxa may not be seen interacting during sampling). In practice, observed interactions often serve as proxies for realised interactions. However, this distinction becomes important when detection biases are strong, as is frequent in many datasets containing rare species (Catchen et al. 2023). False negatives in interaction data can impact the inference of ecological networks, and accounting for observation errors in predictive models can mitigate this issue (Catchen et al. 2023). For example, Weinstein and Graham (2017b) developed a hierarchical model for hummingbird‐plant interactions that explicitly accounts for detection probability, with was estimated under 25% in their dataset. Observed and realised interactions may thus differ considerably, and local interactions may arise from both the ecological and sampling processes taking place locally.

Local networks are defined within a particular location and time. Even though space and time are continuous variables that should yield probability densities of interactions (i.e., relative likelihoods of interactions occurring at infinitesimal locations and instants in time), they can be treated as spatial patches and time segments. The spatial boundary of local networks may be delineated by a collection of geographic coordinates $\left(x,y,z\right)$, with $\left(x,y\right)$ representing longitude and latitude coordinates, and z denoting altitudes or depths. Ecological interactions may vary along latitudinal and altitudinal gradients, as evidenced by changes in hummingbird‐plant interactions (Weinstein and Graham 2017a, 2017b) and mosquito biting rates (e.g., Kulkarni et al. 2006) at different elevations. On the other hand, time is treated as the specific time period within which interactions were observed or predicted. Treating space and time as discrete dimensions aligns with the common sampling methods of ecological networks and provides probabilities of interactions, which can be obtained by integrating probability densities over space and time. We can quantify both an area (or volume) $A_{0}$ and a duration $t_{0}$ with these definitions. The choice of area and duration when sampling local networks depends on the study system, the ecological processes we aim to capture, and available resources (e.g., Olesen et al. 2008 sampled an Arctic pollination network daily over two seasons to study network assembly). By sampling and modelling local networks, we may thus conduct spatiotemporal analyses of interactions (Box 1, Figure 1).

### 3.2 What Are Local Probabilistic Interactions Conditioned on?

#### 3.2.1 Local Interactions May Be Conditioned on Co‐Occurrence

The probability that two taxa i and j interact in a local network $L_{x,y,z,t}$ (spatial and temporal subscripts hereafter replaced by k for clarity) can be conditioned on different variables. In addition to network area (or volume) $A_{0}$ and duration $t_{0}$, they may be conditioned on taxa co‐occurrence $X_{i,j,k}$, which describes if the geographic distributions of both taxa overlap within the study area. As illustrated in Box 1, co‐occurrence may be modelled probabilistically, in which case it may conform to a Bernoulli distribution $X_{i,j,k}∼\text{Bernoulli}\left(\varphi_{i,j,k}\right)$, where $\varphi_{i,j,k}=P\left(X_{i,j,k}=1\right)$. The probability of co‐occurrence can be calculated using the marginal occurrence probabilities $P\left(X_{i,k}=1\right)$ and $P\left(X_{j,k}=1\right)$. Given that taxa occurrences are not independent of each other, the probability of co‐occurrence can be calculated as follows:

(1) $$P\left(X_{i,j,k}\right)=P\left(X_{i,k},X_{j,k}\right)=P\left(X_{i,k}|X_{j,k}\right)\times P\left(X_{j,k}\right).$$

Note that for concision, the probability notation used in this manuscript implicitly assigns a value of 1 to binary variables (e.g., in Equation (1), $P\left(X_{i,k}|X_{j,k}\right)$ is short for $P\left(X_{i,k}=1|X_{j,k}=1\right)$), unless stated otherwise. The value is stated explicitly when it is 0 or when emphasising the value of 1.

The probability of co‐occurrence $P\left(X_{i,j,k}\right)$ can be estimated using joint species distribution models (e.g., Pollock et al. 2014), potentially taking into account biotic interactions (Staniczenko et al. 2017). Given that the probability that two non‐co‐occurring taxa interact locally is zero (i.e., $P\left(L_{i,j,k}=1|X_{i,j,k}=0\right)=0$), the probability of local interaction can be obtained as follows:

(2) $$P\left(L_{i,j,k}=1\right)=P\left(L_{i,j,k}=1|X_{i,j,k}=1\right)\times P\left(X_{i,j,k}=1\right).$$

Knowing if two taxa co‐occur improves our estimation of the probability that they interact locally by mitigating a potential source of uncertainty.

#### 3.2.2 Local Interactions May Be Conditioned on Different Environmental and Biological Factors

Local interactions may also be conditioned on local environmental variables such as temperature (Angilletta Jr. et al. 2004), precipitation (Woodward et al. 2012), habitat structure (Klecka and Boukal 2014) and the presence or abundance of other taxa (Pilosof et al. 2017; Kéfi et al. 2012). We use the variable $E_{k}$ as a placeholder for the set of environmental variables used for estimation. For example, in Gravel et al. 2019, $E_{k}$ represents two distinct variables: temperature and precipitation. Both can be treated as distinct conditions in the expression of interaction probability. Environmental variables can be binary (e.g., presence of another taxa), discrete (e.g., abundance of another taxa), or continuous (e.g., temperature and precipitation). Like co‐occurrence, $E_{k}$ can also be probabilistic when considering the variability or uncertainty of environmental factors. $E_{k}$ represents all environmental variables that were considered when measuring interaction probabilities; it is therefore a subset of all environmental factors actually impacting ecological interactions.

Other factors impacting interaction probabilities locally are taxa local abundances $N_{i,k}$ and $N_{j,k}$, which affect encounter probabilities (Canard et al. 2012), and local traits $T_{i,k}$ and $T_{j,k}$ (e.g., movement rates, Beardsell et al. 2021; Cherif et al. 2024), which may impact encounter probabilities and the ability of individuals to interact after encountering each other (Bartomeus et al. 2016; Caron et al. 2024; Poisot et al. 2015). Local interaction probabilities may also be conditioned on higher‐level properties such as network structure, which we denote by $f\left(L_{k}\right)$. Many topological null models (i.e., statistical models that randomise interactions by retaining certain properties of the network while excluding others) provide interaction probabilities from selected measures of network structure, such as connectance (Fortuna and Bascompte 2006) and the degree distribution (Bascompte et al. 2003). Like $E_{k}$, the variables $T_{i,k}$, $T_{j,k}$ and $f\left(L_{k}\right)$ are placeholders for more specific biological variables, which need to be clearly identified when documenting local interaction probabilities.

#### 3.2.3 Local Interactions May Be Conditioned on Biological Feasibility

Local interactions must be biologically feasible before occurring at a specific time and space. A local probability of interaction $P\left(L_{i,j,k}\right)$ can be expressed as the product of the probability of local interaction given that the taxa can potentially interact $P\left(L_{i,j,k}=1|M_{i,j}=1\right)$, with their probability of regional interaction $P\left(M_{i,j}=1\right)$:

(3) $$P\left(L_{i,j,k}=1\right)=P\left(L_{i,j,k}=1|M_{i,j}=1\right)\times P\left(M_{i,j}=1\right),$$

assuming that $P\left(L_{i,j,k}=1|M_{i,j}=0\right)=0$.

Low values of $P\left(L_{i,j,k}|M_{i,j}\right)$ indicate that potential interactions rarely occur locally, intermediate values around 50% suggest considerable spatiotemporal variability, while high values indicate that potential interactions almost always occur locally. The local probability of interaction between two taxa is thus always equal or below their probability of regional interaction. Considering biological feasibility when estimating local interaction probabilities leverages information from the metaweb to better predict the local occurrence of interactions (Strydom et al. 2021; Dansereau et al. 2024).

#### 3.2.4 Conditional Variables Must Be Explicitly Stated

The probability that two taxa i and j interact in a local network $L_{k}$ can thus be conditioned on their co‐occurrence $X_{i,j,k}$ (or more explicitly on their occurrences $X_{i,k}$ and $X_{j,k}$), local abundances $N_{i,k}$ and $N_{j,k}$, local traits $T_{i,k}$ and $T_{j,k}$, local environmental conditions $E_{k}$, network area (or volume) $A_{0}$, time interval $t_{0}$, network properties $f\left(L_{k}\right)$ and biological feasibility $M_{i,j}$. When these conditions are absent from an expression, it may be because they have been marginalised over. Interaction probabilities may also be implicitly conditioned on missing variables (e.g., when estimated for specific values of these variables without explicitly including them as conditions), potentially impacting our interpretation. The local probability of interaction is described by the following expression when all conditional variables are included:

(4) $$P\left(L_{i,j,k}|X_{i,k},X_{j,k},N_{i,k},N_{j,k},T_{i,k},T_{j,k},E_{k},A_{0},t_{0},f\left(L_{k}\right),M_{i,j}\right).$$

**FIGURE 2:**
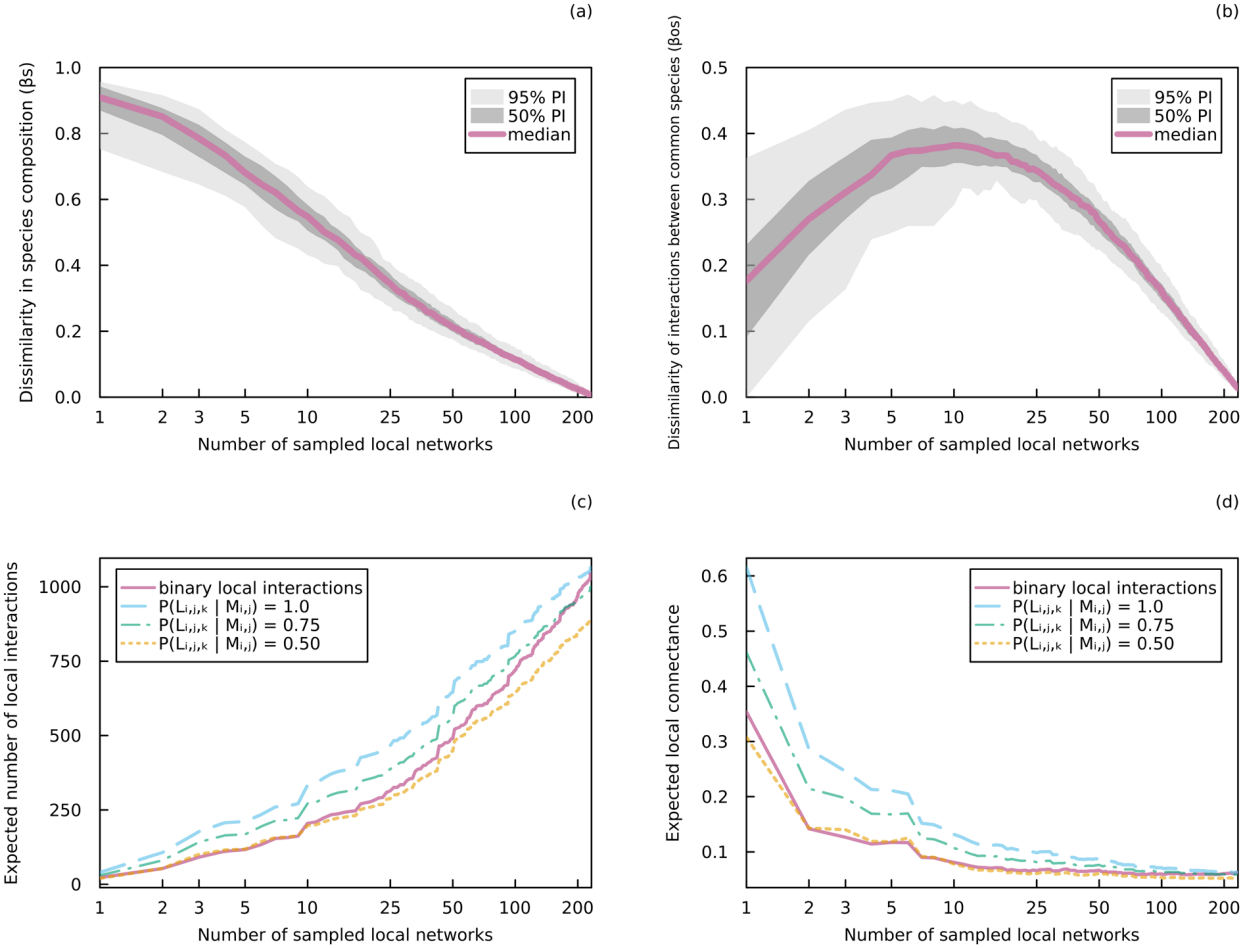
Network accumulation curves. (a) Dissimilarity in species composition and (b) dissimilarity of interactions between common species between aggregated local networks and the metaweb of binary host–parasite interactions. In both panels, the coloured line represents the median dissimilarity across simulations and the grey areas cover the 50% and 95% percentile intervals. (c) Scaling of the number of interactions and (d) scaling of connectance with the number of sampled (aggregated) binary and probabilistic local interaction networks. For a better comparison with binary interactions, local networks of probabilistic interactions were derived from a metaweb of probabilistic interactions with a false positive and false negative rate of zero. A specific value of $P\left(L_{i,j,k}|M_{i,j}\right)$ (the local probability of interaction among potentially interacting species) was used for all non‐aggregated local networks within a particular curve. Aggregated local networks were obtained by sequentially and randomly selecting a number of local networks and aggregating both their species and interactions (with the value of $P\left(L_{i,j,k}|M_{i,j}\right)$ increasing in aggregated local networks of probabilistic interactions). All data are from Kopelke et al. (2017), and more details on the analysis can be found in Box 2 and Data S1.

These conditional variables do not all need to be considered at all times. The ones that should be considered depend on the study system, the objectives of the study, and the resources available to the researchers. For example, Gravel et al. (2019) analysed local European host–parasite networks of willow‐galling sawflies and their natural enemies, all referenced in space and time, to infer local interaction probabilities between co‐occurring species. This was achieved by including temperature and precipitation as conditional variables. In Box 2 and Figure 2, we reuse these data to show the extent of variation among local networks. We do so by measuring their dissimilarity with the metaweb aggregating all interactions, both in terms of species composition and interactions. We estimated local probabilistic interactions following Equation (3), showing that insufficient local variation (high probability of local interaction among potentially interacting species) results in an overestimation in the number of interactions and connectance (i.e., the proportion of non‐forbidden links that are realised). This analysis was conducted for illustrative purposes, and other conditional variables could have been used.

BOX 2 **Dissimilarity of local host–parasite networks.**

Comparing local networks to the metaweb helps us evaluate the local realisation of potential interactions. For instance, Noreika et al. (2019) examined the dissimilarity between local and regional networks to explore whether potential plant‐pollinator interactions are more likely to occur in better‐connected and restored environments. Here we compare local tripartite host–parasite networks to the metaweb to analyse the variability of local networks and their dissimilarity regarding species composition and interactions. We use data from Kopelke et al. (2017), consisting of interactions between willows, willow‐galling sawflies, and their natural enemies sampled in different networks across Europe. All data manipulation and methods are described in Data S1, as well as the formulas used to calculate dissimilarity measures and connectance. All code and data to reproduce these analyses are available on Zenodo (https://doi.org/10.5281/zenodo.15476609).

To simulate different sampling levels, we aggregated local networks of binary interactions by sequentially and randomly selecting a number of networks and aggregating both their species and interactions. We built the metaweb of binary interactions by aggregating all local networks. In Figure 2a,b, we show how the dissimilarity between the metaweb and aggregated local networks changes with the number of sampled networks. We compared the metaweb to the aggregated local networks using the dissimilarity in species composition ($\beta_{S}$, Figure 2a) and the dissimilarity of interactions between common species ($\beta_{\mathrm{OS}}$, Figure 2b) indices (Poisot et al. 2012). Expectedly, local networks are highly dissimilar from the metaweb in terms of species composition, especially when only a limited number of sites have been sampled. This is because few species from the metaweb (species pool) occur locally. Moreover, we observe a peak in the dissimilarity of interactions between common species at intermediate sampling levels. This suggests that species are collected faster than their interactions. With a limited number of sampled local networks, few regional interactions are observed locally. Adding more sites brings new species, but not always their interactions. Quadratic relationships of network properties with sampling effort were also observed by McLeod et al. (2021). This type of analysis can offer insights into the amount of sampling required to get a more comprehensive understanding of the metaweb.

Next, we converted binary regional interactions to probabilistic ones by applying constant false positive and negative rates across all interactions, and predicted local networks of probabilistic interactions using Equation (3) and different values of $P\left(L_{i,j,k}|M_{i,j}\right)$. These local networks were then sequentially and randomly aggregated to investigate how the number of interactions and connectance scale with the number of sampled networks (Figure 2c,d). By comparing the scaling relationships observed in local networks of binary and probabilistic interactions, we observe that high values of $P\left(L_{i,j,k}|M_{i,j}\right)$ lead to systematic overestimations in the number of interactions and connectance, especially when $P\left(L_{i,j,k}|M_{i,j}\right)=1$ (i.e., when local and regional probabilities of interactions are equivalent). This suggests that high values of $P\left(L_{i,j,k}|M_{i,j}\right)$ do not adequately capture the variability of local interactions. However, these biases tend to diminish as the number of sampled networks increases, indicating that most interactions are eventually captured when $P\left(L_{i,j,k}|M_{i,j}\right)$ is high. In contrast, low values of $P\left(L_{i,j,k}|M_{i,j}\right)$ lead to missing interactions, resulting in an underestimation in the number of interactions and connectance when the number of sampled networks is high. These results underscore the importance of using the appropriate level of variability when estimating local interaction probabilities, and show how sampling effort can impact our estimation of the probability that potential interactions occur locally.

When accounted for, conditional variables should be clearly described in the documentation of the data (Brimacombe et al. 2023), preferentially in mathematical terms. For instance, ecologists should be explicit about their consideration ($P\left(L_{i,j,k}|X_{i,j,k}\right)$) or not ($P\left(L_{i,j,k}\right)$) of co‐occurrence, as this can change our interpretation of the data and their uncertainty sources. In Table 1, we present examples of studies that used different conditional variables. This table includes the probability of empirically observing an interaction that is realised locally $P\left(O_{i,j,k}|L_{i,j,k}\right)$ to underscore the distinction between local observations and realisations of interactions.

**TABLE 1:** Mathematical expressions of probabilistic interactions.

Expression	Type	Outcome	Common models	References
$P\left(L_{i,j,k}|X_{i,k},X_{j,k},\ldots \right)$	Local	Realisation of the interaction given taxa co‐occurrence	Species distribution models	Gravel et al. (2019); Dansereau et al. (2024); Boxes 1 and 5
$P\left(L_{i,j,k}|N_{i,k},N_{j,k},\ldots \right)$	Local	Realisation of the interaction given taxa abundances	Neutral models	Canard et al. (2014)
$P\left(L_{i,j,k}|T_{i,k},T_{j,k},\ldots \right)$	Local	Realisation of the interaction given local traits	Trait matching models	Caron et al. (2024); Box 4
$P\left(L_{i,j,k}|E_{k},\ldots \right)$	Local	Realisation of the interaction given local environmental conditions	Environmental‐based models	Gravel et al. (2019) (temperature and precipitation)
$P\left(L_{i,j,k}|A_{0},\ldots \right)$	Local	Realisation of the interaction in a given area or volume	Spatial models	Galiana et al. (2018) *, Box 3
$P\left(L_{i,j,k}|t_{0},\ldots \right)$	Local	Realisation of the interaction during a given time period	Temporal models	Weinstein and Graham 2017a; Boxes 1 and 3
$P\left(L_{i,j,k}|f\left(L_{k}\right),\ldots \right)$	Local	Realisation of the interaction given network structure	Topological models	Fortuna and Bascompte (2006) (connectance); Stock et al. (2017)
$P\left(L_{i,j,k}|M_{i,j},\ldots \right)$	Local	Realisation of the interaction given that it is biologically feasible	Spatiotemporal models	Dansereau et al. (2024); Boxes 2, 3 and 5
$P\left(O_{i,j,k}|L_{i,j,k},\ldots \right)$	Local	Observation of the interaction given that it is realised locally	Sampling models	Catchen et al. 2023
$P\left(M_{i,j}|T_{i},T_{j}\right)$	Regional	Biological feasibility of the interaction given regional traits (non‐forbiddenness)	Trait matching models	Strydom et al. (2022); Box 4
$P\left(M_{i,j}^{*}|T_{i},T_{j},E\right)$	Regional	Ecological feasibility of the interaction given regional traits and environmental conditions	Trait matching and environmental‐based models	This study

*Note:* The probability of interaction between two taxa i and j is interpreted differently in a local network $L_{k}$ of realised interactions, a local network $O_{k}$ of observed interactions, a metaweb M of potential interactions (representing the *biological* feasibility of interactions), and a metaweb $M^{*}$ of potential interactions (representing the *ecological* feasibility of interactions). Each expression emphasises a different conditional variable, the ellipsis serving as a placeholder for other variables not explicitly stated in the expression. The outcome of each of these probabilistic events, along with common models used for estimation, is presented alongside examples of studies that employed them (with specific variables indicated in parentheses, when applicable). These studies show how environmental, biological and interaction data can be used within these models in a practical way. The study marked with an asterisk (*) has been conducted on binary interaction networks. The boxes in our study that discuss these expressions are also specified.

### 3.3 How Are Local Probabilistic Interactions Estimated?

Interaction matrices derived from direct field observations can be used to estimate local interaction probabilities. When networks are sampled repeatedly across time or space, the proportion of networks where an interaction occurs can serve as an estimation of its variability. This proportion may represent the probability that the interaction occurs in a network similar to those previously sampled. Environmental and biological variables can be used as predictors of binary interactions to enhance our estimations of local interaction probabilities. For example, a generalised linear model with environmental covariates can be used to predict local interaction probabilities (Gravel et al. 2019). Moreover, interaction data can be aggregated into a metaweb to predict local interaction probabilities, as demonstrated in Data S1 using host–parasite interaction data from Kopelke et al. (2017). In the absence of interaction data, environmental and biological factors can still provide valuable predictions of interaction probabilities (e.g., neutral model of Canard et al. 2014). These predictions must, however, be validated with empirical data to assess model quality. Examples of probabilistic models of local interactions are provided in Table 1. Several studies have used raw interaction data to fit or validate these models (Table 1). These studies can serve as practical examples for ecologists looking to integrate raw interaction data into probabilistic models. For a deeper discussion of the challenges and opportunities in the modelling of species interactions, refer to Strydom et al. (2021).

When using multiple competing models to estimate local interaction probabilities, rather than selecting a single model that best fits the data, model averaging may enhance our estimations. Model weights represent the probability that each model is the most suitable for explaining the data. They can be obtained using Akaike weights, a measure derived from the Akaike information criterion based on prediction error (Burnham and Anderson 2004; Wagenmakers and Farrell 2004). For instance, given two competing models $\mathrm{mo}d_{1}$ and $\mathrm{mo}d_{2}$ with respective probabilities $P\left(\mathrm{mo}d_{1}\right)$ and $P\left(\mathrm{mo}d_{2}\right)$, interaction probability can be calculated as follows:

(5) $$P\left(L_{i,j,k}\right)=P\left(L_{i,j,k}|\mathrm{mo}d_{1}\right)\times P\left(\mathrm{mo}d_{1}\right)+P\left(L_{i,j,k}|\mathrm{mo}d_{2}\right)\times P\left(\mathrm{mo}d_{2}\right).$$

Model averaging takes into account the uncertainty of model structure. In addition to model structure, it is crucial to quantify and disclose all sources of uncertainty to better understand the validity and limitations of our predictions of local interactions (Simmonds et al. 2024).

## 4 Metawebs: Regional Catalogues of Interactions

### 4.1 What Are Regional Probabilistic Interactions?

Metawebs (Dunne 2006) are networks of potential interactions over broad spatial, temporal and taxonomic scales, which is why they are referred to as regional networks. They are the temporal and spatial asymptotes of local interactions (Box 1, Figure 1). Over time, two co‐occurring taxa should eventually interact in at least one location with suitable environmental conditions if their traits can support an interaction, and will never interact otherwise. Potential interactions thus describe the biological capacity of taxa to interact under suitable environmental conditions if they are given enough time to do so.

Metawebs have been used for different types of direct interactions, such as predator–prey (e.g., Maiorano et al. 2020), host–parasite (e.g., Gravel et al. 2019) and plant‐pollinator (e.g., Aguiar et al. 2024) interactions. For instance, they were used to investigate the effects of connectivity and restoration efforts in plant‐pollinator networks (Noreika et al. 2019) and evaluate sampling completeness in avian frugivory networks (Martins et al. 2022). This was achieved by analysing the proportion of potential interactions realised locally, which is typically lower (i.e., high dissimilarity between regional and local networks, Box 2, Figure 2) when local interactions are more context‐dependent and environmentally driven. Metawebs of probabilistic interactions are particularly useful when there is uncertainty in the ability of taxa to interact (Strydom et al. 2023). They may also be used as priors of local interactions (i.e., first estimates of local interaction probabilities, as done in Dansereau et al. 2024), which can then be updated with local data to obtain better informed local interaction probabilities (e.g., using Equation 3). Therefore, building a metaweb of probabilistic interactions may be an important first step before predicting local networks.

In contrast to local interactions, where uncertainty arises from the variability of interactions and the lack of information on the conditions, uncertainty in metawebs solely results from a lack of knowledge. This uncertainty arises due to insufficient interaction data, especially for taxa that have not yet been observed to co‐occur, and uncertainties in trait‐matching models. As data accumulate, regional interactions should tend toward binarity, either taking a value of 1 (observing an interaction at least once) or approaching 0 (repeatedly failing to observe an interaction). Confidently observing an interaction once confirms its biological feasibility, but due to the possibility of false negatives, failing to observe it does not necessarily indicate that it is unfeasible (Catchen et al. 2023 show how to estimate the rate of false negatives in ecological networks). While local interaction probabilities are irreducible because of local variability, the uncertainty of regional interactions reduces to 0 with the addition of information. Moreover, although *neutrally* forbidden interactions (i.e., forbidden interactions between rare species, Canard et al. 2012) have low probability in local networks, they would have a probability of 1 in the metaweb (this is because the species' traits could support an interaction if they were to encounter each other at high enough abundances). Likewise, non‐co‐occurring taxa may have a non‐zero probability of interaction in the metaweb. Regional interaction probabilities are thus fundamentally different from local interaction probabilities, both in terms of uncertainty sources and probability values.

**FIGURE 3:**
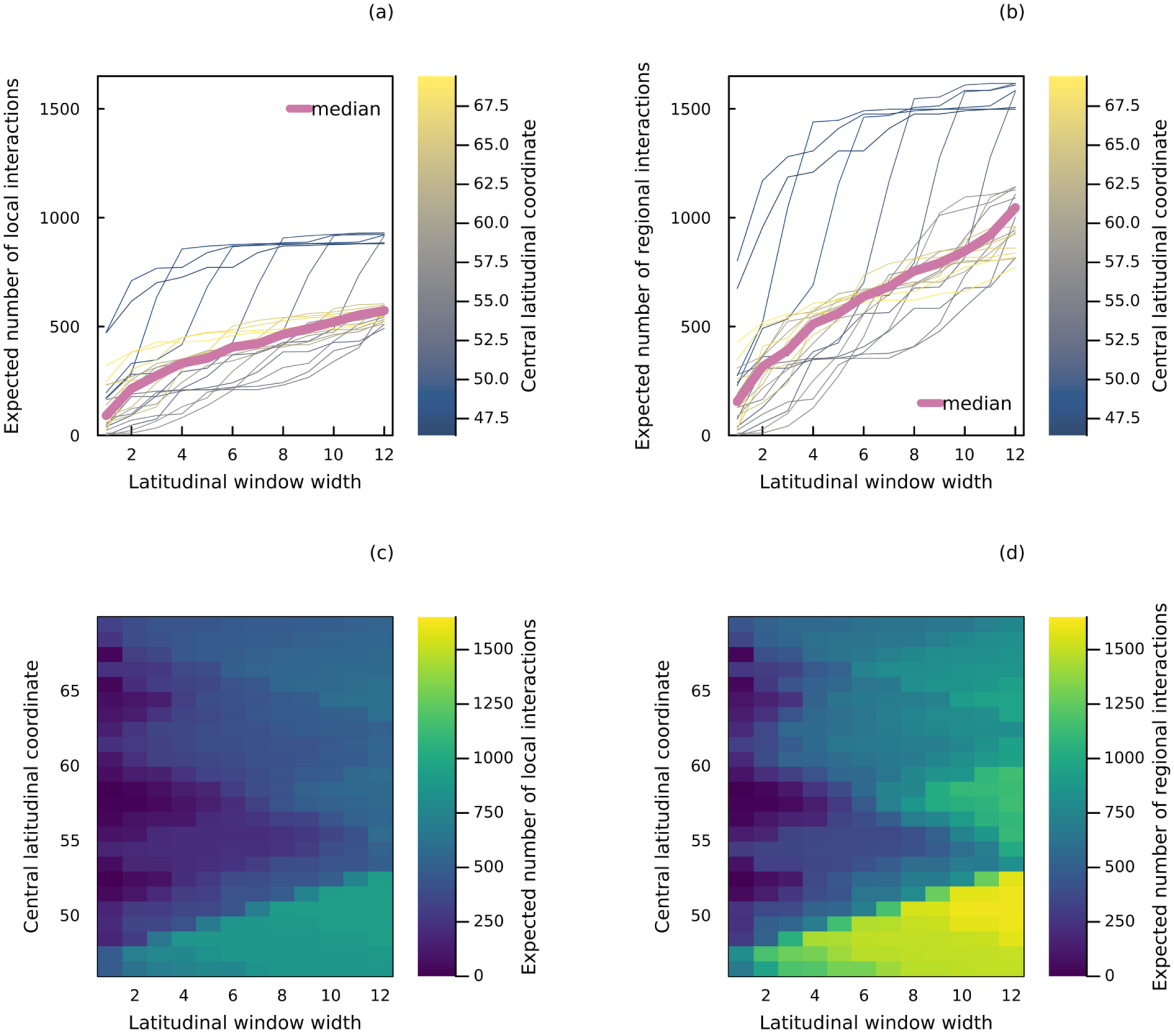
Spatial scaling of interactions. Expected number of host–parasite interactions in a network aggregating all (a) local and (b) regional probabilistic interactions within a latitudinal window of a given width. Every dashed curve corresponds to a different window centered at a given latitude (colour bar), with the pink solid line representing the median number of interactions across windows. Heatmaps of the expected number of (c) local and (d) regional interactions found in windows of specified width and position (central latitude). Probabilities of regional interactions were obtained with a false positive rate of 5% and a false negative rate of 10%. Local probabilistic interactions were derived from regional probabilistic interactions by setting the value of $P\left(L_{i,j,k}|M_{i,j}\right)$ (the local probability of interaction among potentially interacting species) to 1. Aggregated local networks were obtained by aggregating both the species and interactions found within a particular latitudinal window, with the values of $P\left(L_{i,j,k}|M_{i,j}\right)$ remaining at their maximum value of 1. All data are from Kopelke et al. (2017), and more details on the analysis can be found in Box 3 and Data S1.

The extent of sampling effort influences our evaluation of regional interaction probabilities, as more interactions can be captured over a larger area or longer duration (Box 3, Figure 3; McLeod et al. 2021). However, in contrast with local interactions, regional interactions are not evaluated for any particular local context (they are rather a collection of local contexts), which impacts how they scale with space and time (notably through the extent of the region covered and sampling duration). In Box 3, we discuss the differences in spatial and temporal scaling of regional interactions compared to local interactions. We do so by using the host–parasite networks of Kopelke et al. (2017) as an illustration of spatial scaling (Figure 3). Understanding the effect of spatial and temporal scales (including sampling effort) on local and regional interaction probabilities is important for effectively propagating uncertainty across scales.

BOX 3 **Spatial and temporal scaling of interactions.**

Local networks and metawebs scale differently with space (area or volume) and time (sampling effort or duration). Local interaction probabilities scale both spatially and temporally, because local interactions have more opportunities to be realised in larger areas and longer durations. In a larger sampling area and duration, we increase the likelihood of sampling favourable conditions for interactions to occur. If a local network of probabilistic interactions $L_{1}$ with an area $A_{1}$ is compared to a larger network $L_{0}$ with an area $A_{0}$, and $A_{1}$ is entirely nested within $A_{0}$, interaction probabilities should be lower in the smaller network, that is, $P\left(L_{i,j,1}|A_{1}<A_{0}\right)\leq P\left(L_{i,j,0}|A_{0}\right)$. However, if $A_{1}$ and $A_{0}$ are disjoint, interaction probabilities could be higher in the smaller area, contingent upon local environmental and biological conditions. In practice, this can be accounted for by increasing the value of $P\left(L_{i,j,k}|M_{i,j}\right)$ as we scale up spatially or temporally, provided that smaller networks are incorporated into larger ones. In contrast, regional interaction probabilities do not scale with space and time. The probability that two taxa potentially interact should be similar in all metawebs in which they are present regardless of scale, provided that the data and methods used for estimation are consistent. This is because they depend solely on the biological capacity of taxa to interact, regardless of co‐occurrence and local environmental conditions. However, regional interaction probabilities may change, tending to become more definitive, with increased sampling effort. While understanding the temporal scaling of networks is useful for assessing sampling effort (Weinstein and Graham 2017a), studying network‐area relationships enables more accurate predictions of the effect of habitat loss and fragmentation on biological communities (Galiana et al. 2018).

Reusing the host–parasite network data of Kopelke et al. 2017, we built local and regional networks at different spatial scales by aggregating both the species and interactions (local or regional) found within expanding latitudinal windows. In Figure 3, we show how the expected number of local host–parasite interactions scales with the spatial boundary of the network (represented by the latitudinal window) in comparison with regional interactions. The increase in the number of regional interactions is due to the inclusion of more species in a larger area. To ensure a conservative comparison between local and regional interactions, we employed equal interaction probabilities (i.e., using $P\left(L_{i,j,k}|M_{i,j}\right)=1$) in both types of network. This means that local interaction probabilities could not increase further during aggregation. Despite this, we notice that the total number of regional interactions scales more rapidly than local interactions. This is because numerous regional interactions involve species that never co‐occur, and as a result, these interactions are not captured in local networks. Therefore, the spatial scaling of networks is inherently determined by the nature of local and regional interactions, regardless of whether interaction probabilities differ between the two types of networks. All data manipulation and methods are described in Data S1, and all code and data to reproduce these analyses are available on Zenodo (https://doi.org/10.5281/zenodo.15476609).

### 4.2 What Are Regional Probabilistic Interactions Conditioned on?

#### 4.2.1 Regional Interactions Describing Biological Feasibility Are Conditioned on Traits

Potential interactions describe the biological feasibility of interactions, which is based solely on the regional traits distributions $T_{i}$ and $T_{j}$ of taxa i and j. We define regional traits distributions as the range of phenotypes a taxon can express across various environments. Local traits $T_{i,k}$ and $T_{j,k}$, which vary spatially and temporally because of phenotypic plasticity (Berg and Ellers 2010), are a subset of regional traits. In practice, $T_{i}$ and $T_{j}$ may correspond to summary measures of trait distributions (e.g., the average body mass of a species). The probability of potential interaction in a metaweb M describing biological feasibility may be expressed as:

(6) $$P\left(M_{i,j}|T_{i},T_{j}\right),$$

which, in contrast with local networks, is not conditioned on any spatial, temporal, co‐occurrence or environmental variables (Table 1). Because phylogenetically close species often share similar traits, closely related species often have similar interacting partners. We can thus use phylogenetic trees to predict species traits and infer regional interactions (Strydom et al. 2022; Eklöf and Stouffer 2016; Stouffer et al. 2012). The taxonomic level of interactions influences the distribution of regional traits. However, as explained in Box 4, there is no fundamental difference in the taxonomic scaling of regional and local interactions (i.e., how interaction probabilities change with taxonomic level) because they both depend on trait aggregation.

BOX 4 **Taxonomic scaling of interactions.**

Given that our interpretation of the properties of ecological networks depends on their taxonomic level (Melián et al. 2011), investigating the taxonomic scaling of interactions (i.e., how interaction probabilities change with taxonomic level) is important. There are no inherent differences between the taxonomic scaling of local and regional interactions. The taxonomic level of interactions impacts the definition of nodes, and local and regional interaction probabilities are not directly conditioned on taxonomic scale. However, some conditional variables (e.g., trait distribution) may covary with taxonomic scale. In such cases, local and regional interaction probabilities would change taxonomically following the scaling of these variables. A better understanding of the taxonomic scaling of local and regional interactions could help us generate networks at the right level of organisation for addressing specific ecological and evolutionary questions (Guimarães 2020), even when network data are collected at a different taxonomic resolution.

In both types of networks, transitioning to a broader level of organisation (e.g., from a species‐level network S to a genus‐level network G) can be done using interaction probabilities from finer scales. For example, in a network with $n_{1}$ species of genus $g_{1}$ and $n_{2}$ species of genus $g_{2}$, one can calculate the probability that at least one species from genus $g_{1}$ interacts with at least one species from genus $g_{2}$ (i.e., the probability that the genus‐level interaction occurs) as follows:

(18) $$P\left(G_{g_{1},g_{2}}\right)=1-\prod_{i=1}^{n_{1}}\prod_{j=1}^{n_{2}}\left(1-P\left(S_{g_{1,i},g_{2,j}}\right)\right),$$

where $g_{1,i}$ and $g_{2,j}$ are the species of the corresponding genus. This equation (which provides the complement probability that no species pair interacts) assumes independence between species‐level interactions, which may not hold true in practice due to the strong phylogenetic signal frequently encountered in species interactions (Gomez et al. 2010). In contrast, a different approach is necessary when transitioning from a broader to a finer level of organisation. This is because knowing that an interaction between two genera occurs does not guarantee that all possible pairwise species combinations will also interact. One possible method is to build a finer‐scale network by generating probabilities of interaction through random sampling from a beta distribution, parameterized by the broader‐scale network.

Fundamentally, the taxonomic scaling of interactions involves aggregating interactions between individuals into larger groups. Interaction probabilities at broader taxonomic scales should thus conform to probabilities of interactions between individuals. For example, Canard et al. (2012) built a species‐based network using simulated individual‐based networks. In local individual‐based food webs, the probability that two individuals interact reflects our degree of belief that one individual will consume the other. Likewise, in local species‐based food webs, the probability that two species interact represents our degree of belief that *at least* one individual from the predator species will consume at least another individual from the prey species. In that regard, taxonomic scaling is analogous to the spatial and temporal scaling of interactions, as they all represent different ways to aggregate individuals into broader groups (either spatially, temporally, or taxonomically).

The biological feasibility of interactions represents the probability that there exists at least one combination of phenotypes that could support a specific type of interaction if they were to encounter each other, assuming they had enough time to interact. This probability is evaluated without incorporating environmental variables in the model. It is the complement of the probability $P\left(F_{i,j}|T_{i},T_{j}\right)$ of forbidden interactions (i.e., the probability that their traits do not support an interaction), which is based uniquely on biological traits:

(7) $$P\left(M_{i,j}|T_{i},T_{j}\right)=1-P\left(F_{i,j}|T_{i},T_{j}\right).$$

For example, let i be a western diamondback rattlesnake ( *Crotalus atrox* Baird and Girard, 1853) and j, a wood lemming ( *Myopus schisticolor* Lilljeborg, 1844). These two taxa never co‐occur, the rattlesnake being adapted to warm regions of North America (Castoe et al. 2007) and the lemming, to northern habitats of Eurasia (Fedorov et al. 2008). As we lack observations of an interaction between these species, we have to rely on expert knowledge or trait‐matching models to estimate their probability of potential interaction. To accurately estimate this probability, we need to ensure that the set of traits considered reflects the overall traits distributions of both taxa. We could for instance consider their average body mass and the average phylogenetic distance of lemmings to rattlesnakes' prey. Doing so, we might find a high probability of potential interaction. This example illustrates how regional interactions may be estimated solely based on traits, without taking into account environmental conditions.

#### 4.2.2 Regional Interactions Describing Ecological Feasibility Are Conditioned on Traits and Environmental Conditions

The biological feasibility of interactions should not be confused with their ecological feasibility. The probability of potential interaction in a metaweb $M^{*}$ describing ecological feasibility may be expressed as:

(8) $$P\left(M_{i,j}^{*}|T_{i},T_{j},E\right),$$

where E is a set of environmental variables (Table 1). Unlike $E_{k}$, these variables do not represent environmental conditions occurring at specific locations. Ecological feasibility represents the probability that two taxa interact if they were to encounter each other under given environmental conditions, assuming they had enough time to interact. Incorporating environmental conditions into a trait‐matching model may be important when there is high covariance between the environment and traits. In our example involving rattlesnakes and lemmings, the probability of potential interaction may be low in most environmental conditions. Western diamondback rattlesnakes are unactive under low temperatures (Kissner et al. 1997), whereas wood lemmings have low tolerance to high temperatures (Kausrud et al. 2008). The probability that an interaction is ecologically feasible is always lower than the probability that it is biologically feasible, even across all environmental conditions:

$$P\left(M_{i,j}^{*}|T_{i},T_{j}\right)=\int_{E}P\left(M_{i,j}^{*}|T_{i},T_{j},E\right)g\left(E|T_{i},T_{j}\right)\mathrm{dE}$$

(9) $$\leq P\left(M_{i,j}|T_{i},T_{j}\right),$$

where $g\left(E|T_{i},T_{j}\right)$ is the conditional probability density function of E given $T_{i}$ and $T_{j}.$.

This is because the biological feasibility of an interaction is a prerequisite for its ecological feasibility. In other words, biological feasibility is necessary but not sufficient for an interaction to be ecologically feasible. Our discussion of metawebs focuses on the biological feasibility of interactions since most methods developed for inferring regional interactions do not explicitly consider environmental conditions (e.g., Strydom et al. 2022).

### 4.3 How Are Regional Probabilistic Interactions Estimated?

Starting from a selected set of taxa distributed within a region of interest, metawebs can be built using different data sources, including literature review (e.g., Maiorano et al. 2020), aggregated interaction data (e.g., Gravel et al. 2019; Saravia et al. 2022), trait‐matching models (e.g., Shaw et al. 2024; Strydom et al. 2022) and expert knowledge. Every pair of taxa confidently observed to interact at least once can be given a probability of 1 because we know they *can* interact. This differs from local interactions, where interaction events may remain stochastic (i.e., $P\left(L_{i,j,k}\right)<1$) even after empirically observing interactions due to their variability. Interactions that were never observed typically have low probability values in local networks and vary from low to high values in metawebs, reaching 0 for forbidden links. The aggregation of observations and predictions tends to raise the number of potential interactions in metawebs.

When using local interaction data to estimate probabilities of regional interactions, repeatedly failing to observe an interaction decreases the probability that it is biologically feasible. Using Bayes' theorem, the probability that the interaction is biologically feasible given that it was never observed locally, $P\left(M_{i,j}=1|O_{i,j,k}=0\right)$, may be calculated as follows:

(10) $$P\left(M_{i,j}=1|O_{i,j,k}=0\right)=\frac{P\left(O_{i,j,k}=0|M_{i,j}=1\right)\times P\left(M_{i,j}=1\right)}{P\left(O_{i,j,k}=0\right)}.$$

The reduction in the probability of regional interaction after considering the lack of observations (i.e., $P\left(M_{i,j}=1|O_{i,j,k}=0\right)<P\left(M_{i,j}=1\right)$) occurs because $P\left(O_{i,j,k}=0|M_{i,j}=1\right)$ must be lower than $P\left(O_{i,j,k}=0\right)$, that is, there is a higher chance of observing an interaction when we know it is biologically feasible.

Observations of interactions may be false positives because of errors due to taxonomic misidentifications and ecological misinterpretations, such as those involving phylogenetically close species or cryptic interactions (Pringle and Hutchinson 2020). Likewise, forbidden interactions may be false negatives if they were evaluated based on unrepresentative or incomplete traits distributions. Employing Bayesian models proves valuable when estimating regional interaction probabilities (e.g., Bartomeus et al. 2016; Cirtwill et al. 2019) by updating prior information (e.g., expert knowledge of interaction probabilities) with empirical data on interactions and traits. By improving our estimation of potential interaction probabilities, we may build more reliable metawebs that better reflect our uncertainty on the biological feasibility of interactions.

## 5 Future Perspectives

**FIGURE 4:**
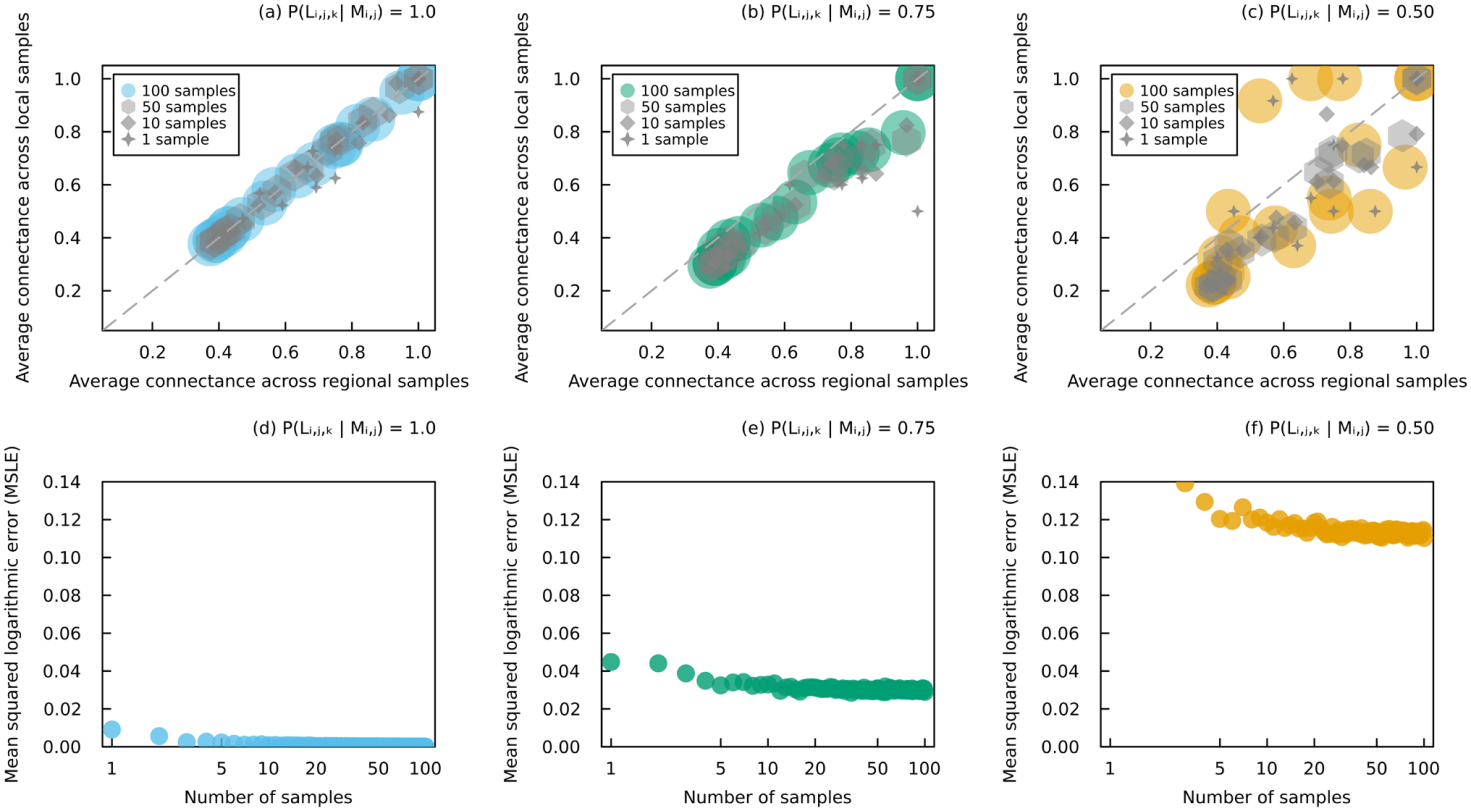
Connectance of sampled binary interaction networks. (a–c) Average connectance of binary interaction networks obtained from the two sampling techniques for 20 randomly selected host–parasite networks. Cross markers represent the connectance of a single sample for each network, diamond markers the average connectance across 10 samples, hexagon markers the average connectance across 50 samples, and the coloured circles the average connectance across 100 samples (marker size proportional to the number of samples). (d‐f) Reduction in the mean squared logarithmic error between the average connectance of binary interaction networks (all 233 host–parasite networks) obtained from these two sampling techniques as the number of samples increases. The local probability of interaction between potentially interacting species was set to three different values: (a, d) $P\left(L_{i,j,k}|M_{i,j}\right)=1.0$, (b, e) $P\left(L_{i,j,k}|M_{i,j}\right)=0.75$, and (c, f) $P\left(L_{i,j,k}|M_{i,j}\right)=0.50$. Probabilities of regional interactions were obtained with a false positive rate of 5% and a false negative rate of 10%. Regional samples were obtained by randomly sampling binary interactions from the probabilistic interaction metaweb, and then propagating this result to all local networks that include the species potentially engaged in the interactions. Local samples were obtained by independently sampling binary interactions for each local network of probabilistic interactions. All data are from Kopelke et al. (2017), and more details on the analysis can be found in Box 5 and Data S1.

In this contribution, we underline the importance of network documentation for adequately interpreting and manipulating probabilistic interaction data. Clear documentation should describe the type of interaction (local or regional) and the conditions under which they were estimated. We show that local networks and metawebs differ in their spatial and temporal scaling (Box 3, Figure 3), with regional interactions remaining consistent across scales. In contrast with metawebs, local interactions are evaluated in a specific context (e.g., in a given area, time and biological and environmental conditions) and depend on co‐occurrence. These differences highlight the need to use probabilistic data with caution, for instance when generating network realisations across space (Box 5, Figure 4). Here we identify key research priorities for improving our understanding of probabilistic local and regional interactions.

BOX 5 **Sampling for binary interaction networks.**

Probabilistic interactions are valuable for assessing the uncertainty of interactions, but we often rely on thresholding or sampling to convert probabilities into binary predictions. These approaches can be employed, for example, to validate models, analyse network structure, or support decision making. Local networks of binary interactions may be predicted by performing independent Bernoulli trials for each probabilistic interaction. This can be particularly useful when analysing the structure of probabilistic interaction networks in the absence of specific analytical formulas (Poisot et al. 2016), even though it may introduce biases in our estimations when connectance is low (Poisot and Gravel 2014; Chagnon 2015). There are at least two techniques to sampling binary interaction networks across space, each predicting a different network for each location within a given region. The first technique performs a single Bernoulli trial for each pair of taxa based on their regional probability of interaction:

(19) $$M_{i,j}∼\text{Bernoulli}\left(\varphi_{i,j}\right),$$

where $\varphi_{i,j}=P\left(M_{i,j}=1\right)$.

In employing this technique, we predict a single metaweb of binary interactions for each simulation. Every pair of taxa predicted to interact in this metaweb will be treated as interacting in all localised networks where they co‐occur, i.e., $L_{i,j,k}=M_{i,j}$ when $X_{i,j,k}=1$. This will result in local pairwise interactions without spatial variation.

The second technique is to independently sample each local network of probabilistic interactions:

(20) $$L_{i,j,k}∼\text{Bernoulli}\left(\varphi_{i,j,k}\right),$$

where $\varphi_{i,j,k}=P\left(L_{i,j,k}=1\right)$.

This can be achieved by first generating distinct probabilistic interaction networks for each location. Because binary interactions are sampled independently for each location, this second technique captures network structure across space and time more effectively. When sampling binary interactions from local interaction probabilities, it is crucial to sample at the same spatial scale for which probabilities were estimated to prevent systematic biases in predictions.

In Figure 4, we compare the average connectance of binary interaction networks resulting from these two sampling techniques. We sampled regional and local interactions from our host–parasite networks of probabilistic interactions (derived from the original data of Kopelke et al. 2017), generating a number of binary interaction network realisations for each site in the dataset. These two sampling techniques yield different outcomes, particularly for intermediate values of $P\left(L_{i,j,k}|M_{i,j}\right)$ of 0.50, which represent instances where regional interactions do not consistently manifest locally (i.e., with the largest local variability). As anticipated, we observe that sampling binary interactions from the metaweb tends to overestimate connectance on average compared to sampling them from local networks (Figure 4). The magnitude of this overestimation depends on the value of $P\left(L_{i,j,k}|M_{i,j}\right)$, with differences in connectance being larger when this probability is small, that is, when local interaction probabilities are much lower than regional ones. We also observe an increase in the variability of connectance when employing a single simulation (Figure 4a–c, cross markers), which is a more tangible representation of the ecological process leading to the realisation of local interactions in nature. All data manipulation and methods are described in Data S1, and all code and data to reproduce these analyses are available on Zenodo (https://doi.org/10.5281/zenodo.15476609).

Both sampling techniques assume independence between interactions, which may not hold true in reality. The realisation of interactions can be influenced by other interactions (Golubski and Abrams 2011) or the presence and abundance of other taxa (Pilosof et al. 2017; Kéfi et al. 2012). For example, a predator may consume a particular prey only if preferred prey are not readily available. The consequences of assuming independence when predicting network structure have yet to be empirically examined. Different approaches can be used to relax this assumption. One option is to condition interaction probabilities on the occurrence of other interactions. In this case, the interactions that serve as conditions must be sampled first to determine the appropriate probability of interaction. Another approach is to directly sample whole networks instead of pairwise interactions (Battiston et al. 2020). This would require estimating the probability of different network structures, a concept further discussed in the conclusion.

### 5.1 Predicting Local Networks From Metawebs

Metawebs can be used to predict local networks across time and space. Local networks of binary interactions can be reconstructed by selecting a subset of taxa and interactions from the metaweb (Dunne 2006). Selecting taxa can be achieved empirically (e.g., observed occurrence data) or numerically (e.g., species distribution models). As species composition is arguably easier to sample and predict than interactions, the primary challenge lies in deciding which interactions to select from the metaweb. Inferring the structure of local networks from the metaweb before predicting their interactions could hold promise (Strydom et al. 2021), considering that the structure of local networks is constrained by the metaweb (Saravia et al. 2022).

Inferring local networks of probabilistic interactions from a metaweb comes with its own challenges. For example, Dansereau et al. (2024) inferred spatially‐explicit food webs from a metaweb of probabilistic interactions. Their predicted localised food webs are downscaled versions of the metaweb (i.e., localised metawebs with interaction probabilities identical to those in the regional metaweb). To infer local networks of realised interactions, local interaction probabilities must be smaller than regional ones. Inferring local networks from a metaweb by maintaining identical interaction probabilities introduces systematic biases, as discussed in Box 2 (unless networks are seen as downscaled metawebs).

As suggested by McLeod et al. 2021, metawebs establish an upper limit for local interactions (similarly for metawebs of probabilistic interactions, Strydom et al. 2023). The probability of local interaction is lower than the probability of regional interaction, regardless of the variables considered:

(11) $$P\left(L_{i,j,k}|\ldots \right)\leq P\left(M_{i,j}|T_{i},T_{j}\right).$$

Moreover, the probability of regional interaction between two taxa must be higher than the probability of them interacting at any location and time because they may never co‐occur. Specifically, the marginal probability of local interaction across all spatial, temporal and environmental conditions must be less than the probability of regional interaction, that is,

(12) $$\int_{E_{k}}\int_{A_{0}}\int_{t_{0}}P\left(L_{i,j,k}|E_{k},A_{0},t_{0}\right)g\left(E_{k},A_{0},t_{0}\right)\;dt_{0}\;dA_{0}\;dE_{k}\leq P\left(M_{i,j}|T_{i},T_{j}\right),$$

where $g\left(E_{k},A_{0},t_{0}\right)$ is the joint density function of $E_{k}$, $A_{0}$ and $t_{0}$.

Estimating more precisely the probability $P\left(L_{i,j,k}|M_{i,j}\right)$ that two taxa interact locally if they can potentially interact can improve our predictions of local networks from the metaweb. This task is challenging due to the variability of this probability across space, time and species pairs. Using simple models of $P\left(L_{i,j,k}|M_{i,j}\right)$, as shown in Data S1, represents an initial step toward reconstructing local networks from metawebs.

### 5.2 Validating Models and Reducing Uncertainty

Field data are essential for validating predictive models and updating previous estimates. One approach to model validation is to evaluate different probabilistic thresholds that distinguish between predicted interactions and non‐interactions (Cirtwill and Hambäck 2021; Strydom et al. 2022). By comparing these predictions with empirical data, we can identify the most accurate threshold and assess model performance. At the local scale, in addition to validating interactions, we can also compare predicted network structure and interaction variability with empirical measures (e.g., frequency of plant‐pollinator interactions measured through repeated sampling). Validation helps determine whether the model is appropriate for making predictions or requires modifications. Furthermore, new observations allow us to continuously update our predictions. At the regional scale, observing a new interaction increases its probability to 1, while failing to observe it decreases the probability. At the local scale, new observations allow us to adjust previous estimates of interaction variability. This process, often implemented using Bayesian statistics, iteratively refines a model's predictions to better match observed data.

While sampling communities decreases knowledge uncertainty by accumulating evidence for the feasibility and local realisation of interactions, interaction variability cannot be reduced with additional data. Regional interactions should become more definitive (probabilities approaching 0 or 1) as we investigate various conditions, including different combinations of species traits. In comparison, owing to environmental heterogeneity, there will invariably be instances in which a local interaction occurs and others in which it does not, across different times and locations. Quantifying and partitioning all sources of uncertainty will enable us to make more accurate predictions about ecological interactions at various spatial and temporal scales and to identify priority sampling locations to reduce this uncertainty. This will prove to be of vital importance as our time to understand nature runs out, especially at locations where the impacts of climate change and habitat loss hit harder.

### 5.3 Relaxing the Independence Assumption

Estimating local interaction probabilities independently for each taxa pair and assembling them into a network comes with limitations. Predicting local networks of binary interactions based on these probabilities assumes independence between interactions, a condition seldom respected in practice (Golubski and Abrams 2011). The occurrence of an interaction may depend on the realisation of other interactions or the presence or abundance of other taxa (Pilosof et al. 2017; Kéfi et al. 2012). Relaxing this assumption is the next step in the stochastic representation of interactions.

A more accurate representation of the uncertainty and variability of ecological networks involves creating *probabilistic networks* ($P\left(L_{k}\right)$ and $P\left(M\right)$), rather than networks of *probabilistic interactions* ($P\left(L_{i,j,k}\right)$ and $P\left(M_{i,j}\right)$). Probabilistic networks describe the probability that a particular network of binary interactions (its whole adjacency matrix) is realised. For example, Young et al. (2021) employed a Bayesian method to estimate the probability of different plant‐pollinator network structures. Generating probabilistic networks could lead to more accurate predictions of local networks of binary interactions by bypassing the independence assumption. Probabilistic networks could serve as an alternative to null hypothesis significance testing when comparing the structure of a local network to some random expectations or the metaweb (Pellissier et al. 2018, Box 2, Figure 2). These random expectations are typically derived by performing a series of Bernoulli trials on probabilistic interactions, assuming independence, to generate a distribution of networks of binary interactions (Poisot et al. 2016). One could instead compare the likelihood of an observed network to the one of the most likely network structure to directly obtain a measure of discrepancy of the empirical network. Generating probabilistic ecological networks represents a tangible challenge, one that, in the coming years, promises to unlock doors to more advanced and adequate analyses of ecological networks.

## Author Contributions

F.B. conducted the analyses and led the writing of the manuscript. D.G. and T.P. provided guidance on the analyses and interpretation of the results. All authors participated in the conceptualisation, writing and revision of the manuscript.

## Peer Review

The peer review history for this article is available at https://www.webofscience.com/api/gateway/wos/peer‐review/10.1111/ele.70161.

## Supporting information


Data S1.

## Data Availability

All data and code used in this manuscript are permanently archived at: https://doi.org/10.5281/zenodo.15476609.
