# Supplementary material for: Deciphering Probabilistic Species Interaction Networks
Source: Ecol Lett. 2025 Jun 26;28(6):e70161. doi: 10.1111/ele.70161 (PMC12200232; doi:10.1111/ele.70161)
Supplement: Supplementary file 1 — Data S1. [file ELE-28-0-s001.pdf]

Data manipulation and analysis

Appendix 1 to *Deciphering probabilistic species interaction networks*

Francis Banville [1,2,3] Tanya Strydom [1,3] Penelope S. A. Blyth [4] Chris Brimacombe [5] Michael Catchen [3,6] Gabriel Dansereau [1,3] Gracielle Higino [2] Thomas Malpas [4] Hana Mayall [4] Kari Norman [1] Dominique Gravel [2,3] Timothée Poisot [1,3]

[1] Université de Montréal [2] Université de Sherbrooke [3] Quebec Centre for Biodiversity Science [4] University of Sheffield [5] University of Toronto [6] McGill University

This work is released by its authors under a CC-BY 4.0 license

Last revision: *December 16, 2024*

1 Host-parasite network data

1.1. Data description We use the collection of tripartite host-parasite networks sampled across Europe of Kopelke *et al.* (2017). This dataset contains well-resolved binary local interactions between willows (52 species), willow-galling sawflies (96 species), and their parasitoids (126 species). Out of a total of 374 local networks, we retained those containing at least 5 species, resulting in a set of 233 georeferenced local networks (networks sampled within areas of 0.1 to 0.3 km² during June and/or July spanning 29 years). Given its replicated networks spanning large spatiotemporal scales, this dataset is well-suited for analyzing network variability.

We built a metaweb of binary interactions by aggregating all local interactions, which gave us a regional network composed of 274 species and 1080 interactions.

1.2. Metawebs of probabilistic interactions We converted these binary regional interactions into probabilistic ones using simple assumptions. Our aim is not to estimate precise probability values, but to create plausible metawebs of probabilistic interactions for our illustrative examples.

We created two metawebs of probabilistic interactions by employing constant false positive and false negative rates for all regional interactions. In the first metaweb, we set both false positive and false negative rates to zero to prevent artificially inflating the total number of interactions, enabling a more accurate comparison with binary interaction networks. This gave us a probability of regional interaction of 1 when at least one interaction has been observed locally and of 0 in the absence of any observed interaction between a given pair of species. This metaweb was used in Box 2.

In the second metaweb, we introduced a 5% false positive rate to account for spurious interactions and a 10% false negative rate to address the elevated occurrence of missing interactions in ecological networks (Catchen *et al.* 2023). We believe these rates represent reasonable estimates of missing and spurious potential interactions, but confirming their accuracy is challenging due to the unavailability of data on the actual feasibility of interactions. Observed interactions were thus given a probability of regional interaction of 95%, whereas unobserved ones were assigned a probability of 10%. This metaweb was used in Boxes 3 and 5.

1.3. Local networks of probabilistic interactions We built local networks of probabilistic interactions using the taxa found in the empirical local networks and attributing pairwise interaction probabilities based on the metawebs of probabilistic interactions $P(M_{i,j})$ (short for $P(M_{i,j} = 1)$) and a constant value of $P(L_{i,j,k}|M_{i,j})$ (short for $P(L_{i,j,k} = 1|M_{i,j} = 1)$) across interactions:

$$P(L_{i,j,k}) = P(L_{i,j,k}|M_{i,j}) \times P(M_{i,j}). \quad (1)$$

We set all values of $P(L_{i,j,k}|M_{i,j})$ to 0.5, 0.75, or 1.0 depending on the simulation. Intermediate values of $P(L_{i,j,k}|M_{i,j})$ around 50% indicate considerable spatiotemporal variability, while higher values close to 1.0 indicate that regional interactions are nearly always realized locally.

2 Additional methods for Box 2: Dissimilarity of local host-parasite networks

2.1. Dissimilarity between local networks and the metaweb We aggregated local networks of binary interactions by sequentially and randomly selecting a number of local networks and aggregating both their species and

interactions.

We compared the metaweb of binary interactions and the aggregated local networks of binary interactions using the dissimilarity in species composition β_S, and the dissimilarity of interactions between common species β_{OS} indices. Both dissimilarity indices were calculated based on the number of items shared by the two networks (c_{LM}) and the number of items unique to the metaweb (u_M) and the aggregated local network (u_L). The β_S dissimilarity index uses species (nodes) as items being compared, while the β_{OS} index assesses dissimilarity based on interactions between shared species. Both indices were calculated following the β_W index of Whittaker (1960):

$$\beta_W = \frac{c_{LM} + u_L + u_M}{(2c_{LM} + u_L + u_M)/2} - 1.$$

We repeated the aggregation process one hundred times and highlighted the median dissimilarity values across simulations, as well as the 50% and 95% percentile intervals.

2.2. Aggregation of local networks of probabilistic interactions We aggregated local networks of probabilistic interactions similarly to the networks of binary interactions, with the distinction that we also adjusted the value of $P(L_{i,j,k})$ (short for $P(L_{i,j,k} = 1)$) when sampling networks. The constancy of the probability of regional interaction across the entire study area means that any rise in the probability of local interaction is solely attributable to an increase in $P(L_{i,j,k}|M_{i,j})$. We adjusted the value of $P(L_{i,j,k}|M_{i,j})$ as follows. Let L_1 and L_2 be two local networks and $L_{1,2}$ the aggregated network. If $P(L_{i,j,1}|M_{i,j})$ and $P(L_{i,j,2}|M_{i,j})$ are the probabilities that two potentially interacting taxa interact respectively in L_1 and L_2, the probability $P(L_{i,j,1,2}|M_{i,j})$ that these taxa interact in the aggregated network $L_{1,2}$ is obtained by:

$$P(L_{i,j,1,2}|M_{i,j}) = 1 - [1 - P(L_{i,j,1}|M_{i,j})] \times [1 - P(L_{i,j,2}|M_{i,j})], \quad (2)$$

assuming independence between the interaction of the two taxa in different networks. This equation represents the probability that the interaction is realized in either (1) exclusively the local network L_1, (2) exclusively the local network L_2 or (3) both, given that the two taxa have the biological capacity to interact.

We then calculated the probabilities of local interaction of the aggregated networks using eq. 1. The value of $P(L_{i,j,k}|M_{i,j})$ for each curve in Figure 2 (panels c-d) is the probability before aggregating networks.

2.3. Calculation of the expected number of local interactions and connectance We investigated how the number of local interactions and connectance scale with the number of sampled (aggregated) local networks. We calculated the expected numbers of interactions by taking the sum of all binary or probabilistic interaction values. Connectance was calculated as the ratio of the expected number of interactions to the number of possible (non-forbidden) interactions. Because our networks are tripartite, connectance was calculated as follows:

$$Co = \frac{I}{S_S S_G + S_G S_P}, \quad (3)$$

where I is the expected number of interactions, S_S the number of Salix species, S_G the number of galler species, and S_P the number of parasitoid species in the network.

Additional methods for Box 3: Spatial and temporal scaling of interactions

3.1. Aggregation of local and regional networks of probabilistic interactions Local probabilistic interactions were derived from probabilistic regional interactions by setting the value of $P(L_{i,j,k}|M_{i,j})$ (the local probability of interaction among potentially interacting species) to 1, ensuring a conservative comparison between aggregated local networks and metawebs. Aggregated local and regional networks were obtained by aggregating both the species and interactions found within a particular latitudinal window. The values of $P(L_{i,j,k}|M_{i,j})$ in local networks remained at their maximum value of 1 following Eq. 2. Latitudinal windows had different positions (central latitudes) and widths (latitudinal widths).

3.2. Calculation of the expected number of interactions We calculated the expected number of local and regional interactions by taking the sum of all probabilistic interaction values of the aggregated networks.

4

Additional methods for Box 5: Sampling for binary interaction networks

4.1. Sampling using regional interaction probabilities We sampled for binary interaction networks across space, predicting a binary interaction network for each location in our dataset. We performed a single Bernoulli trial for each pair of taxa based on their regional probability of interaction:

$$M_{i,j} \sim \text{Bernoulli}(\phi_{i,j}),$$

where $\phi_{i,j} = P(M_{i,j} = 1)$.

Every pair of taxa predicted to interact in this metaweb will be treated as interacting in all localized networks where they co-occur, i.e. $L_{i,j,k} = M_{i,j}$ when $X_{i,j,k} = 1$.

We performed between 1 and 100 simulations for each location to get a distribution of networks of binary interactions sampled using regional interaction probabilities.

4.2. Sampling using local interaction probabilities We sampled binary interaction networks across space, predicting a binary interaction network for each location in our dataset. We first generated distinct probabilistic interaction networks for each location. The local probability of interaction between potentially interacting species was set to three different values: $P(L_{i,j,k}|M_{i,j}) = 1.0$, $P(L_{i,j,k}|M_{i,j}) = 0.75$, and $P(L_{i,j,k}|M_{i,j}) = 0.50$. We then sampled each local network of probabilistic interactions independently:

$$L_{i,j,k} \sim \text{Bernoulli}(\phi_{i,j,k}),$$

where $\phi_{i,j,k} = P(L_{i,j,k} = 1)$.

We performed between 1 and 100 simulations for each location to get a distribution of networks of binary interactions sampled using local interaction probabilities.

4.3. Calculation of connectance We calculated the connectance of our predicted tripartite networks of binary interactions following eq. 3. We calculated the average connectance across simulations for each location.

4.4. Calculation of the mean squared logarithmic error (MSLE) The mean squared logarithmic error was calculated as follows:

$$MSLE = \frac{\sum(log(\overline{Co_L}) - log(\overline{Co_M}))^2}{n},$$

where $\overline{Co_L}$ and $\overline{Co_M}$ are the average connectance across simulations for each location, respectively for local and regional samples, and n is the number of locations.

References

Catchen, M.D., Poisot, T., Pollock, L.J. & Gonzalez, A. (2023). The missing link: Discerning true from false negatives when sampling species interaction networks.

Kopelke, J.-P., Nyman, T., Cazelles, K., Gravel, D., Vissault, S. & Roslin, T. (2017). Food-web structure of willow-galling sawflies and their natural enemies across Europe. *Ecology*, 98, 1730–1730.

Whittaker, R.H. (1960). Vegetation of the Siskiyou Mountains, Oregon and California. *Ecological Monographs*, 30, 279–338.
